# Factors influencing the pre-hospital management of civilian burn mass casualty incidents in the 21st century: a scoping review

**DOI:** 10.1186/s13049-025-01380-9

**Published:** 2025-05-01

**Authors:** Andreas Lindquist, Resha Al-Azzawi, Torsten Risør, Lasse Raatiniemi

**Affiliations:** 1 Department of Clinical Medicine, Faculty of Health Science, UiT The Arctic University of Norway, Pb 6050 Langnes, N-9037 Tromsoe, Norway; 2 Department of Community Medicine, Faculty of Health Science, UiT The Arctic University of Norway, Tromsoe, Norway; 3 Medical Education Unit, Faculty of Health Science, UiT The Arctic University of Norway, Tromsoe, Norway; 4 Section for General Practice & Research Unit for General Practice Zealand, Department of Public Health, University of Copenhagen, Copenhagen, Denmark; 5 Section for General Practice, Department of Community Medicine, UiT The Arctic University of Norway, Tromsoe, Norway; 6 Department of Air Ambulance, University Hospital of North Norway, Tromsoe, Norway

**Keywords:** Burn mass casualty incidents, Mass casualty incidents, Emergency services, Emergency response, Response strategy, Pre-hospital management, Disaster management, Preparedness planning

## Abstract

**Background:**

Burn mass casualty incidents (BMCI) are unique and catastrophic events that are uncommon but recurring and comprehensively challenge all emergency services involved. The causes range from forces of nature to accidental or intentional explosions, indoor fires and chemical burns. A growing population, climate change exacerbated fire weather, increasing industrial activity and a rising threat of worldwide transnational terrorism all increase the risk of BMCIs. Emergency response strategies are thus of critical importance and can be improved upon by learning from previous incidents through the identification of recurrent themes.

**Objectives:**

Identify, categorise, and describe key themes and factors reported as having a favourable or detrimental influence on the professional management of civilian BMCIs.

**Materials and methods:**

A scoping review following the Arksey and O’Malley framework with enhancements by Levac, Colquhoun and O’Brien, and PRISMA-ScR, was conducted using six electronic databases, including a search for grey literature from January 2001 to March 2024. A total of 51 documents, containing descriptions, discussions, and/or experiences of the pre-hospital management of burn mass casualty incidents in civilian, non-war settings, were included and analysed using thematic analysis for qualitative data and labelled for themes and factors.

**Results:**

Thirteen key themes and 71 factors were identified to influence the pre-hospital management of BMCIs. The key themes were *Command, Communication, Contextual, Education, Environment, Evacuation, Fortuity, Human Factors, Preparedness, Response Tactics, Safety, Triage,* and *Volunteer*. The 71 identified factors were for example self-evacuation, varied non-medical transport methods, traffic congestion and decontamination.

**Conclusion:**

The identified themes and factors provide insights from real-life incidents on what is reported to influence the situation at hand. The identified factors can be used to target specific areas for further improvement in future BMCIs, particularly in preparedness planning and training, for example by taking self-evacuation into account in future disaster plans.

**Supplementary Information:**

The online version contains supplementary material available at 10.1186/s13049-025-01380-9.

## Background

Burn mass casualty incidents (BMCI) are unique and catastrophic events that are uncommon but recurring and comprehensively challenge all emergency services involved [1–3]. There is no consensus for one definition of mass casualty incidents (MCIs) or BMCIs. An incident can be defined as mass casualty when the available healthcare resources, or their management systems are severely challenged or unable to meet the healthcare needs of the affected population [2]. Potential causes for civilian BMCIs vary from disastrous fires in residential buildings, night clubs, hotels, and venues [4–11], to transportation disasters [12–15], industrial accidents [16–22], terrorist attacks [23–25] volcanic eruptions [26], earthquakes [27, 28], and wildfires [29].

BMCIs can rapidly overwhelm whole geographical regions or continents and their specialized burn units [3]. For example, Denmark, Finland and Norway can each handle nationally up to 25 burns patients simultaneously. However, once the number of burns patients in one country exceeds 8, the Nordic BMCI response mechanism is activated, and excess patients are evacuated to neighbouring Nordic countries to ensure no country exceeds its capacity for high-quality care [30]. In Europe, a demand for a similar mechanism has been presented and implemented after a BMCI in Romania in 2015 [3, 31].

On the background of a globally growing population, climate change exacerbated fire weather, and a rising threat of worldwide transnational terrorism, emergency response strategies for BMCIs are hence of critical importance and can be improved upon by learning from previous incidents. Since every BMCI is unique, the experiences from these incidents, both positive and negative, give us valuable experience-based information on how to better prepare and respond to future events.

To our knowledge, there are no published articles that per today gather all reported BMCIs from the 21st century into one comprehensive paper and try to identify common themes and factors. Several reviews focus on BMCIs, but these primarily address in-hospital treatment rather than pre-hospital management, or report generally on BMCIs, often focusing on injury type and/or outcome. Understanding the pre-hospital factors is important for developing more effective response strategies and improving outcomes of future BMCIs.

This paper aims to address this gap by reviewing the available documents and providing evidence-based insights. It identifies, categorizes, and describes key themes and factors perceived as favourable or detrimental for the situation, highlighting those reported to influence the situation directly or indirectly. Additionally, it outlines areas for future research and response planning.

## Methods

This scoping review follows the Arksey and O’Malley framework [32], enhanced by Levac, Colquhoun and O’Brien [33]. The five stages of the framework are detailed as subheadings in this chapter. A conventional double screening for inclusion was conducted (AL, ED) [34, 35]. Due to the scoping nature, a protocol was not prospectively registered.

### The research question

What are the themes and factors reported to directly or indirectly influence the pre-hospital management of BMCIs in civilian settings?

### Identifying relevant literature

A systematic search was conducted in CINAHL, Cochrane Library Trials, Embase, PsycINFO, PubMed, and Web of Science. Grey literature was identified via a systematic Google Scholar search, expert consultations (LR, ER), citation screening of included studies (*n* = 154), and open searches in Google Scholar and the library database for UiT—The Arctic University of Norway. Search strategies are detailed in Supplementary file 1.

### Selection of included literature

*Time period*: Documents published between January 1st, 2001, and April 1st, 2024. The time period was chosen due to its modern nature, reflecting technological advancements in pre-hospital care.

*Inclusion criteria*: Qualitative documents—such as case reports, retrospective analyses, and descriptive studies—that described, discussed, or detailed experiences of pre-hospital management of civilian BMCIs by professional emergency responders. The Population, Concept, and Context (PCC) framework was used.

*Population*: Certified medical emergency first responders in pre-hospital care who provide initial healthcare at an incident or manage it remotely; such as dispatchers, emergency medical technicians (EMTs), paramedics, physicians, and medically trained rescue workers such as firefighters and helicopter emergency medical service (HEMS) operators.

*Concept*: Pre-Hospital management. All actions performed or not performed by professional first responders to handle an incident from its onset until all patients reach a primary treatment facility or no longer require medical care.

*Context*: Civilian BMCIs. In this paper, we define BMCIs as events with multiple burn injury patients with any types of burns, such as thermal burns, chemical burns, scalds, and inhalation injuries, and that arise from various sources, including but not limited to, fires, explosions, industrial accidents, and hazardous materials and that overwhelm the response capabilities of the local healthcare and/or emergency services, and are identified as (B)MCI in the system.

*Exclusion criteria*: Documents describing military BMCIs involving soldiers in war or MCIs with secondary or tertiary burns affecting fewer than 10% of patients. Papers focusing on non-professionals experiences, in-hospital management, or secondary evacuations such as transfers to burn centres, purely quantitative studies, guidelines, reviews, response plans, or simulations, or if they were non-retrievable or written in languages other than English, Swedish, Norwegian, Danish, or Finnish. Major incidents, defined as stressing but not overwhelming systems, were excluded based on the definition by Fattah et al. [36, 37].

*Screening and selection process*: Identified literature was imported into Covidence (Veritas Health Innovation, Melbourne, Australia). Titles and abstracts were double screened (AL, ED), and eligibility was assessed by full-text review. Inclusion decisions were consensus based (AL, ED), with unresolved discrepancies (*n* = 3) referred to a third screener (RA-A). The process adhered to The Preferred Reporting Items for Systematic reviews and Meta-Analyses extension for Scoping Reviews (PRISMA-ScR) guidelines and is presented in Table 1.

**Table 1:**
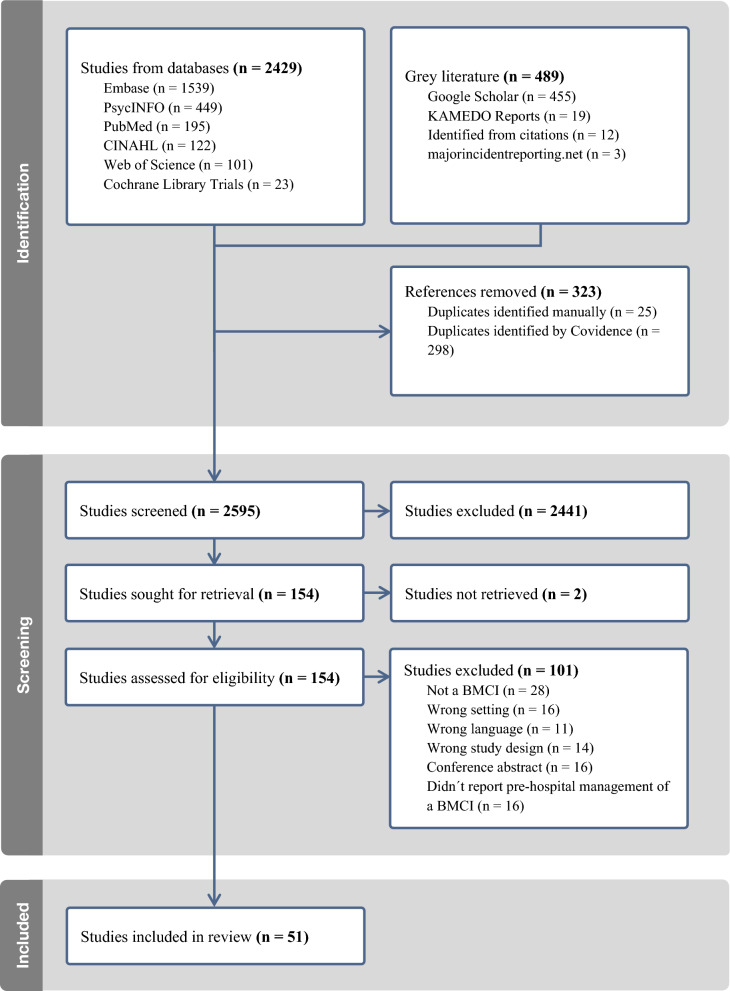
Presents the PRISMA-ScR flowchart outlining the screening and selection process for the included documents

*Undertaking consultation*: Experts in mass casualty incidents (LR) and medical literature (ER) provided inputs to ensure that key literature was identified, leading to all relevant KAMEDO-reports published after 2001 being included for screening.

### Data charting

A data charting template was developed and used in Covidence. First author (AL) reviewed all data and synthesized the extracted information from the included studies into tables for further analyses and labelling. The original data charting template is presented in Supplementary file 2.

### Collating, summarising and reporting results

Thematic analysis was used to refine and label themes and factors from the extracted data [38–40]. To ensure consistency of interpretation and to prevent inter-coder variation, a single analyst (AL) was purposefully chosen to conduct the thematic analysis. This allowed for greater depth of immersion in the data, facilitating nuanced insights and a coherent thematic narrative.

The themes were not pre-determined but emerged inductively from the data. To strengthen the trustworthiness of the analysis, we employed iterative analyses, peer validation, collaborator feedback, a consistency check (intra-rater reliability), and documentation [38–41]. In the iterative analyses, the extracted data was repeatedly revisited and refined, until no new themes or factors were identified and saturation was reached, leading to final themes and factors being labelled and described consistently. Peer validation involved discussing emerging themes with an external peer (ED), who was not involved in data extraction, to challenge assumptions and ensure interpretative consistency. Collaborator feedback was continuously received from co-authors (RA-A, TR, LR), allowing further critique of the themes and supporting accuracy in the thematic interpretation. As an addition to peer validation, written feedback was received in the final stage of refinement (AW, TW), which further pushed the analytical process and contributed to the finalisation of the thematic structure. The iterative process was documented to provide an audit trail for transparency and enhance the trustworthiness of the findings. Finally, a consistency check was conducted by re-coding 10% of the data nine months later and comparing it with the original labelling. This yielded the same results, supporting the trustworthiness and reproducibility of the analysis.

Finally, the identified factors were categorized and counted, with corrections made for duplicate reporting across documents. However, if a factor was reported multiple times within a single document or incident for different reasons, each occurrence was counted separately.

## Results

A total of 2595 citations were screened, resulting in 51 documents [5, 8, 17, 20, 22, 29, 42–86]. These documents reported on 37 unique BMCIs, of which sixteen were classified primarily as indoor fires, seven as industrial incidents, one as an outdoor incident, ten as transportation incidents, and three as wildfires. Among the included documents, 34 were case reports (including 14 retrospective analyses, one descriptive study, one retrospective cohort study, and one analysing injury outcomes). The remaining 17 included three retrospective cohort studies, three analytical reports, three commentaries, two case studies with retrospective analyses, and one each of cross-sectional, observational, retrospective observational, quality improvement, and empirical research studies. The included documents are presented in Supplementary file 3.

From this literature, 13 key themes and 71 unique factors were identified. The key themes represent the overarching areas for potential improvement in BMCIs management, while the factors highlight specific elements that directly or indirectly were reported to influence the response management and can potentially be targeted more precisely for improvement.

The identified key themes were *Command, Communication, Contextual, Education, Environment, Evacuation, Fortuity, Human Factors, Preparedness, Response Tactics, Safety, Triage,* and *Volunteer*, and are presented in Tables 2 and 3. Within these themes, 71 unique factors were identified, each given a descriptive label and categorised accordingly, presented in Table 4. Where applicable, factors were classified by type, using descriptive attributes such as effective, inadequate, sufficient, etc., if reported in the original documents. If no type was specified, only the frequency of the factor was stated. The 71 factors are presented alongside their associated key theme and type(s) in Table 5. In Table 6 we present the included incidents together with their respective name, classification and key statistics.

**Table 2:**
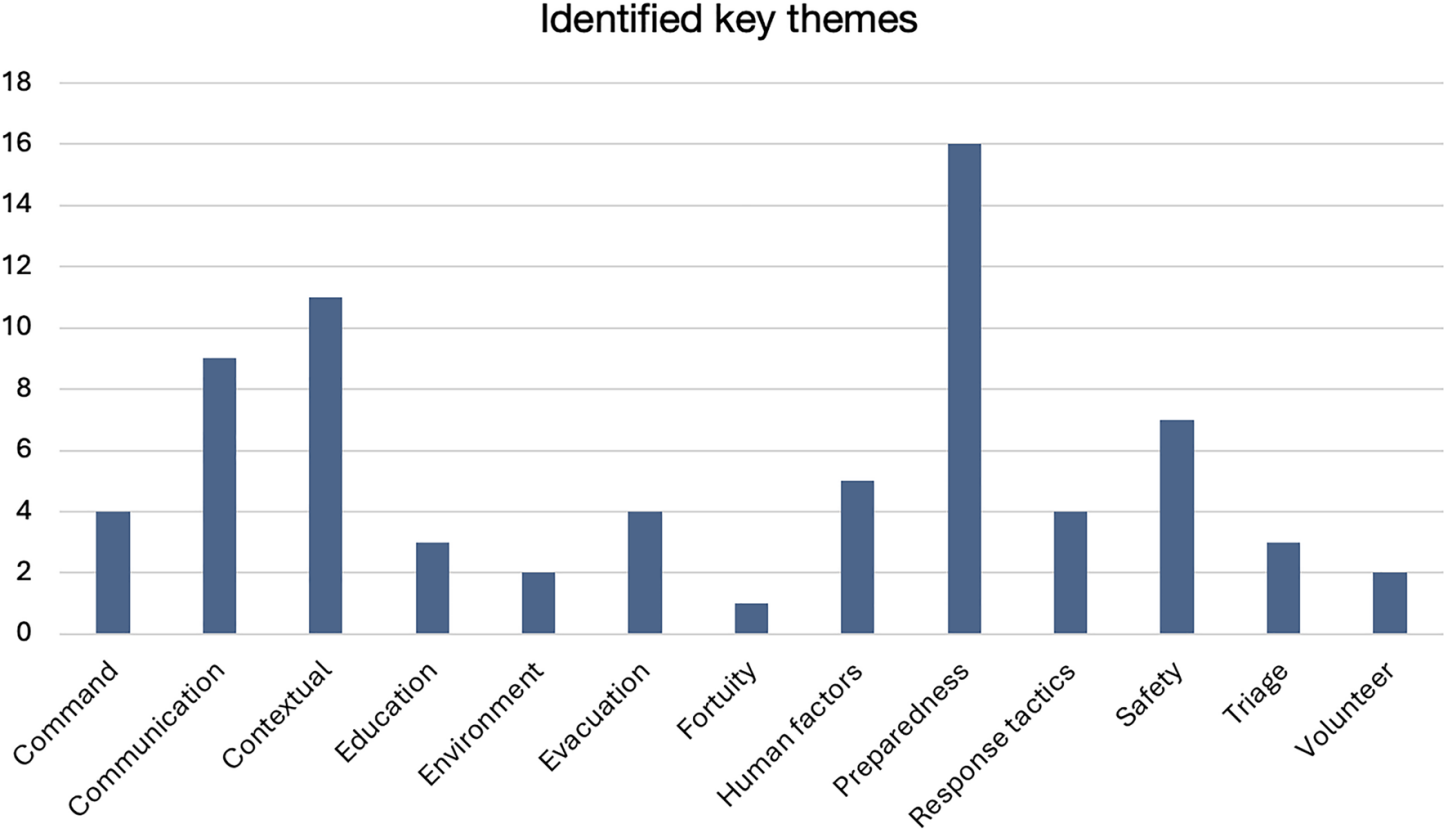
Presents the 13 identified key themes together with the frequency of how often each key theme was assigned to one of the 71 identified factors

**Table 3:**
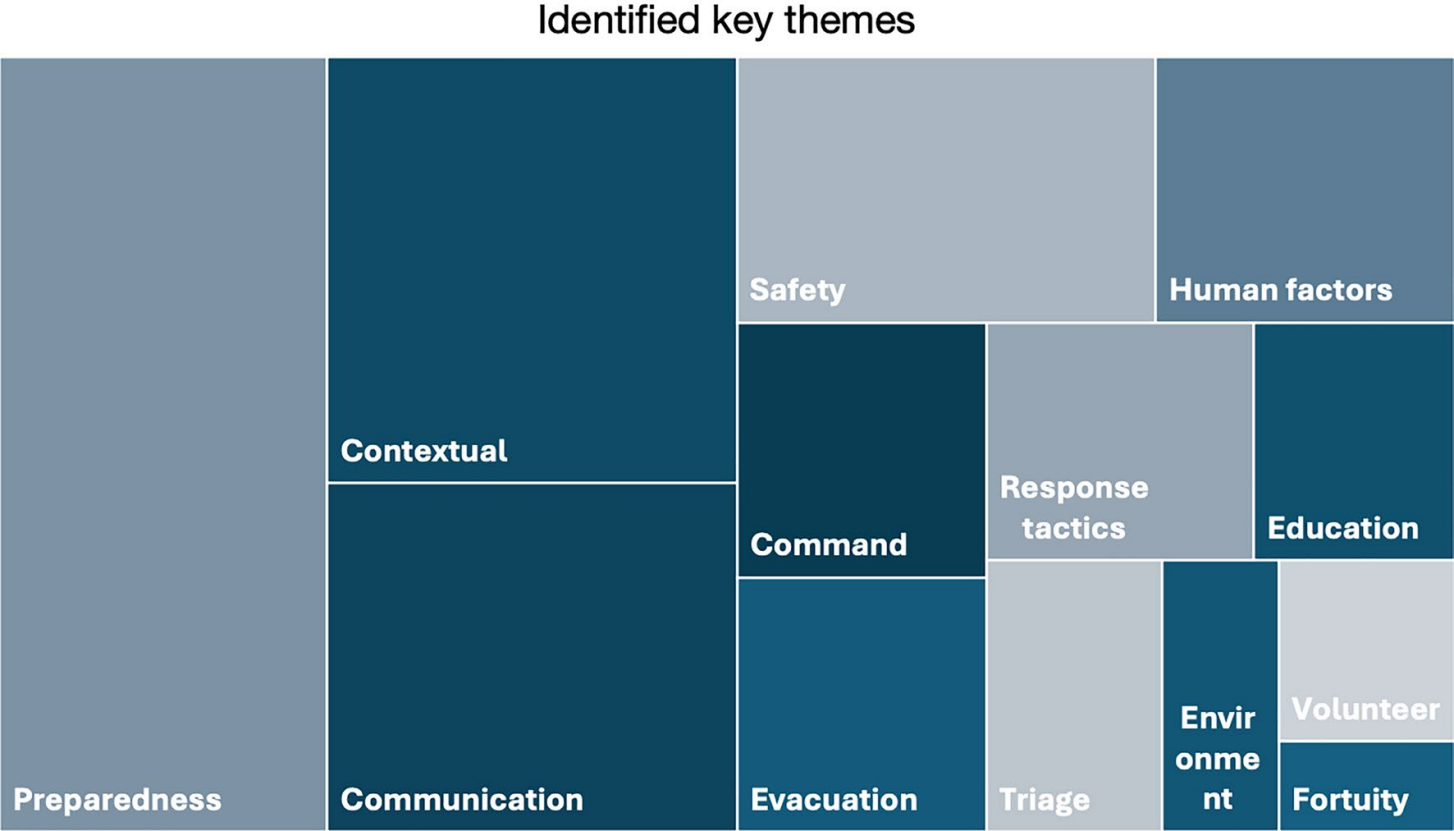
Visually presents the 13 identified key themes, with each theme’s proportional area reflecting its reported frequency to enable a more intuitive interpretation

**Table 4:**
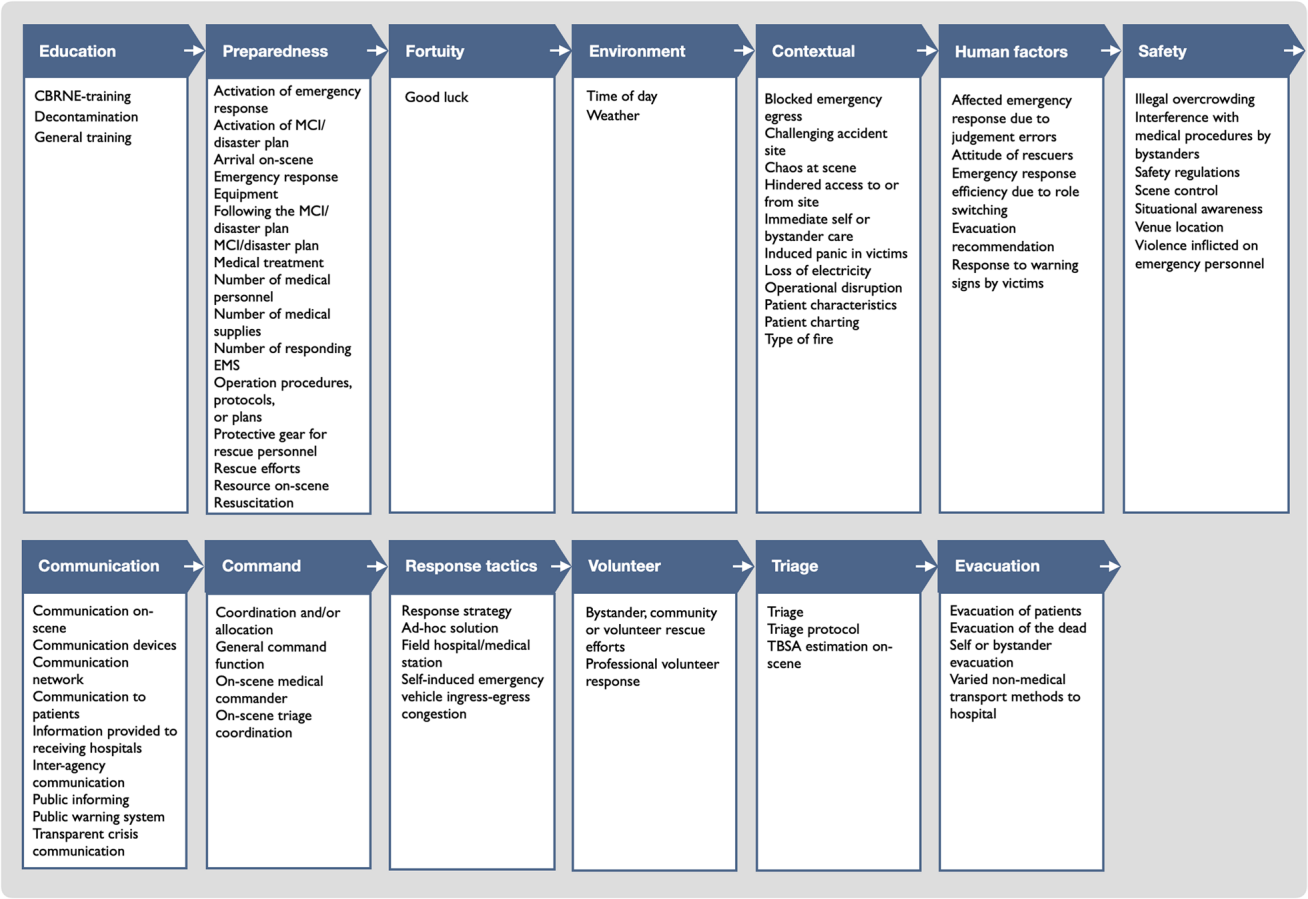
Presents the identified key themes and factors in an operational sequence of a fictional BMCI, illustrating how these elements may emerge in a real-world scenario. This table simplifies the complex interrelationships between themes and factors, providing a visual representation of potential intervention points. For a more detailed description, including classification and frequency of each factor, see Table 5

**Table 5:** The identified factors organised with their most reported key theme and type

Key theme	Identified factor	Type (*n*)
Command	Coordination and/or allocation	Satisfactory (*n* = 7), Unsatisfactory (*n* = 9)
Command	General command function	Effective (*n* = 1), Ineffective (*n* = 4), Satisfactory (*n* = 5), Lack of (*n* = 4)
Command	On-scene medical commander	Lack of (*n* = 4)
Command	On-scene triage coordination	Satisfactory (*n* = 4), Unsatisfactory (*n* = 2), Lack of (*n* = 3)
Communication	Communication on-scene	Satisfactory (*n* = 5), Unsatisfactory (*n* = 10)
Communication	Communication devices	Satisfactory (*n* = 1), Incompatible (*n* = 5), Lack of (*n* = 2)
Communication	Communication network	Stability (*n* = 1), Overload (*n* = 6), Failure (*n* = 8)
Communication	Communication to patients	Satisfactory (*n* = 1)
Communication	Information provided to receiving hospitals	Adequate (*n* = 2), Unsatisfactory (*n* = 4), Lack of (*n* = 3)
Communication	Inter-agency communication	Satisfactory (*n* = 1), Unsatisfactory (*n* = 7), Lack of (*n* = 3)
Communication	Public informing	Effective (*n* = 2), Ineffective (*n* = 1), Hampering (*n* = 1)
Communication	Public warning system	Lack of (*n* = 1)
Communication	Transparent crisis communication	(*n* = 1)
Contextual	Blocked emergency egress	(*n* = 2)
Contextual	Challenging accident site	(*n* = *7*)
Contextual	Chaos at scene	(*n* = 6)
Contextual	Hindered access to or from site	(*n* = 9)
Contextual	Immediate self or bystander care	(*n* = 2)
Contextual	Induced panic in victims	(*n* = 1)
Contextual	Loss of electricity	(*n* = 3)
Contextual	Operational disruption	(*n* = 8)
Contextual	Patient characteristics	(*n* = 5)
Contextual	Patient charting	Adherent (*n* = 1), Deviation from (*n* = 3)
Contextual	Type of fire	Intense (*n* = 1), Rapid (*n* = 2)
Education	CBRNE-training	Insufficient (*n* = 2), Lack of (*n* = 3)
Education	Decontamination	Insufficient (*n* = 2), Lack of (*n* = 2)
Education	General training	Sufficient (*n* = 3), Insufficient (*n* = 6), Non-compliance (*n* = 1), Lack of (*n* = 6)
Environment	Time of day	Daytime (*n* = 1), Night-time (*n* = 4)
Environment	Weather	Good (*n* = 1), Bad (*n* = 3), Cold (*n* = 5)
Evacuation	Evacuation of patients	Timely (*n* = 2), Delayed (*n* = 5), Hampered (*n* = 4), Impossible (*n* = 1)
Evacuation	Evacuation of the dead	(*n* = 2)
Evacuation	Self or bystander evacuation	(*n* = 14)
Evacuation	Varied non-medical transport methods to hospital	(*n* = 17)
Fortuity	Good luck	(*n* = 3)
Human factors	Affected emergency response due to judgement errors	Compromised (*n* = 1)
Human factors	Attitude of rescuers	Poor (*n* = 1)
Human factors	Emergency response efficiency due to role switching	Secured (*n* = 1), Compromised (*n* = 2)
Human factors	Evacuation recommendation	Non-compliance (*n* = 1)
Human factors	Response to warning signs by victims	Inadequate (*n* = 1)
Preparedness	Activation of emergency response	Rapid (*n* = 6), Delayed (*n* = 3)
Preparedness	Activation of MCI/disaster plan	Rapid or immediate (*n* = 6), Delayed (*n* = 1)
Preparedness	Arrival on-scene	Delayed (*n* = 3)
Preparedness	Emergency response	Rapid (*n* = 16), Scalable (*n* = 9), Delayed (*n* = 13), Effective (*n* = 5), Lack of (*n* = 4)
Preparedness	Equipment	Inappropriate or inadequate (*n* = 4), Malfunctioning (*n* = 5), Lack of (*n* = 3)
Preparedness	Following the MCI/disaster plan	Compliance (*n* = 4), Non-compliance (*n* = 2)
Preparedness	MCI/disaster plan	Effective (*n* = 5), Incomplete (*n* = 9), Lack of (*n* = 1)
Preparedness	Medical treatment	Sufficient (*n* = 2), Insufficient (*n* = 2), Lack of (*n* = 1)
Preparedness	Number of medical personnel	Sufficient (*n* = 3), Insufficient (*n* = 3)
Preparedness	Number of medical supplies	Insufficient (*n* = 6)
Preparedness	Number of responding emergency services	Insufficient (*n* = 9), Sufficient (*n* = 8)
Preparedness	Operation procedures, protocols, or plans	Practical (*n* = 1), Impractical (*n* = 2), Non-compliance (*n* = 1)
Preparedness	Protective gear for rescue personnel	Inappropriate or inadequate (*n* = 3)
Preparedness	Rescue efforts	Effective (*n* = 3)
Preparedness	Resource on-scene	Insufficient (*n* = 1), Lack of (*n* = 1)
Preparedness	Resuscitation	Lack of (*n* = 2)
Response tactics	Response strategy	“Load and go” (*n* = 5), “Stay and play” (*n* = 1)
Response tactics	Ad-hoc solution	Emergency shelter (*n* = 4), Triage area (*n* = 3), Medical treatment (*n* = 2), Ambulance overload strategy (*n* = 2), Communication enhancement (*n* = 1), Field hospital (*n* = 1), Triage criteria (*n* = 1)
Response tactics	Field hospital/medical station	Use of (*n* = 4), Lack of (*n* = 1)
Response tactics	Self-induced emergency vehicle ingress-egress congestion	(*n* = 3)
Safety	Illegal overcrowding	(*n* = 2)
Safety	Interference with medical procedures by bystanders	(*n* = 1)
Safety	Safety regulations	Non-compliance (*n* = 3)
Safety	Scene control	Sufficient (*n* = 4), Insufficient (*n* = 6)
Safety	Situational awareness	Insufficient (*n* = 2), Lack of (*n* = *3*)
Safety	Venue location	Inappropriate use (*n* = 2)
Safety	Violence inflicted on emergency personnel	Verbal (*n* = 2), Physical (*n* = 1)
Triage	Triage	Satisfactory or correct (*n* = 9), Unsatisfactory or incorrect (*n* = 8), Not performed (*n* = 3), Rapid (*n* = 2)
Triage	Triage protocol	Adherence to (*n* = 2), Deviation from (*n* = 8), Lack of (*n* = 1)
Triage	TBSA estimation on-scene	Unsuccessful (*n* = 2)
Volunteer	Bystander, community or volunteer rescue efforts	(*n* = 12)
Volunteer	Professional volunteer response	Coordinated (*n* = 2), Uncoordinated (*n* = 2)

Table 5 presents the 71 identified factors in the middle column, alongside their most reported key theme in the left column and their type and frequency (n) in the right column. As shown in the right column, the type of factor varies; some are neutral, indicating only frequency, while others are classified as either positive or negative, such as effective or ineffective, or descriptive attributes such as rapid, poor, or adequate for example. If two or more papers reported the same factor from the same incident, the count was adjusted to ensure that it was recorded only once as a unique identified factor

**Table 6:** Incidents included in the review

Name of Incident	Location of Incident	Year of incident	Classification of incident	Casualties	On scene deaths	Articles included
–	Czech Republic	–	Industrial *Explosion and* *indoor fire at a factory*	18	–	1
Gothenburg discothèque fire	Sweden	1998	Indoor fire *Nightclub*	213	61	3
–	Saudi Arabia	1999	Indoor fire *Tent*	169	37	1
Enschede fireworks disaster	The Netherlands	2000	Industrial *Indoor fire and explosions at a* *factory*	947	21	1
Singapore airlines flight 006: Accident	Taiwan	2000	Transportation *Aviation accident*	179	79	1
Volendam New Years café fire	The Netherlands	2001	Indoor fire *Café*	245	4	2
Terrorist attack on the Pentagon	USA	2001	Transportation *Terrorist attack with airplane*	292	189	2
WTC terrorist attacks	USA	2001	Transportation *Terrorist attack with airplane*	–	2753	2
Bali bombings	Indonesia	2002	Indoor fire *Terrorist attack with bombs*	> 300	180	2
Station Nightclub Fire	USA	2003	Indoor fire *Nightclub*	215	96	3
Canberra Bushfires	Australia	2003	Wildfire	–	–	1
West Pharmaceutical Services explosion	USA	2003	Industrial *Factory explosion*	> 30	3	1
San Diego County Firestorm	USA	2003	Wildfire	138	16	1
Beslan School Seige	Russian Federation	2004	Indoor fire *Terrorist attack with bombs*	> 1000	329	1
–	Pakistan	2004	Indoor fire *Terrorist attack with bombs*	104	14	1
–	China	2005	Industrial *Chemical accident at factory*	118	0	1
Nakumatt Supermarket fire	Kenya	2009	Indoor fire *Supermarket*	–	26	1
-	Kenya	2009	Transportation *Vehicle fire and explosion*	178	91	1
Black Saturday Bushfires	Australia	2009	Wildfire	414	170	2
The Hermosillo ABC daycare fire	Mexico	2009	Indoor fire *Daycare/warehouse*	148	29	1
–	USA	2009	Industrial *Explosion and indoor fire at factory*	68	3	1
–	Chile	2010	Indoor fire *Prison*	466	81	1
–	The Netherlands	2011	Indoor fire *Nursing home*	49	0	1
–	India	2012	Transportation *BLEVE*	41	1	1
Kiss Nightclub Fire	Brazil	2013	Indoor fire *Nightclub*	1002	234	1
Gudvanga tunnel fire	Norway	2013	Transportation *Vehicle and* *tunnel fire*	66	0	1
–	China	2013	Industrial *Chemical accident at factory*	41	10	1
–	China	2014	Transportation *Chemical accident*	253	0	1
Hangzhou bus attack	China	2014	Transportation *Indoor fire in a bus*	33	0	1
–	South Korea	2014	Indoor fire *Hospital*	183	1	1
–	Mexico	2015	Indoor fire *Explosion at hospital*	71	-	1
Formosa Fun Water Park Dust Explosion	Taiwan	2015	Outdoor incident *Dust explosion*	499	0	7
–	Japan	2017	Industrial *Explosion and* *indoor fire in a factory*	11	1	1
Miryang hospital fire	South Korea	2018	Indoor fire *Hospital*	192	37	1
Borgo Panigale explosion	Italy	2018	Transportation *BLEVE*	158	1	1
–	China	2020	Transportation *BLEVE*	176	20	1
–	Italy	2023	Indoor fire *Nursing home*	87	6	1

Table 6 shows all included BMCIs, listing the name of the incident where applicable, the classification of the incident, as well as the number of casualties, on-scene fatalities and how many articles reported on each incident. Notably, the Formosa Fun Water Park Dust Explosion (2015) stands out as the most reported incident, appearing in seven included articles. Indoor fires were the most frequently reported incident type, occurring in nightclubs (e.g., Station Nightclub Fire, Kiss Nightclub Fire), hospitals (e.g., Miryang Hospital Fire), and warehouses (e.g., Gothenburg discothèque fire). The Beslan School Siege had the highest reported casualties (> 1,000), while the World Trade Center (WTC) attacks recorded the most on-scene deaths (2,753). Geographical disparities in BMCI reporting are evident, with most incidents occurring in high-income countries. However, cases from Pakistan, Kenya, China, and India highlight BMCIs as a global phenomenon, though underreporting in some regions may limit available data

## Discussion

We identified 13 key themes, and 71 factors reported to influence the pre-hospital management of civilian BMCIs. The identified key themes represent the overarching areas for potential improvement in BMCIs management, while the factors highlight specific elements that can potentially be targeted more precisely for improvement. Every identified factor is potentially important. The mere fact that a factor has been reported in a previous BMCI makes it significant and worthy of consideration for future response.

In our discussion, we highlight factors we believe may have greater practical relevance. The discussed factors are grouped together, and discussed in the natural flow of an BMCI, aligning with the operational sequence of its management. Factors we consider particularly important are summarised in our conclusion.

### Emergency response

A commonly identified factor was rapid emergency response [20, 22, 44, 47, 51, 53, 63, 65, 67, 69, 73, 74, 76, 85, 86], likely reflecting the robust preparedness and resource availability in high-income countries (HIC), where many of the incidents included in our study took place. In contrast, delayed emergency response also arose in several articles, suggesting more complex reasons than insufficient preparedness alone. Examples included slow hospital-based team deployment [47], lack of knowledge about available resources [55], concerns for responder safety [20], absence of helicopter evacuation [22], and long distances for ambulance support [81].

In certain cases, pre-hospital services were effectively unavailable, identified as lack of emergency response [17, 45, 62, 64]. These incidents occurred in non-Western countries such as Saudi Arabia, Pakistan, India, and Kenya, with at least three in low-income areas lacking established emergency services. Notably, although many burn accidents are known to occur in low- and middle-income countries (LMIC) [87], most articles in this review stem from high-income nations. This mismatch may reflect limited academic output from LMICs, potentially leading to underrepresentation of their experiences and thereby likely limiting the generalizability of our findings.

Differences in emergency response capabilities between low- and high-income countries are well documented in literature [88–90], and further research may help close this gap. As highlighted in the Sendai Framework’s focus on disaster risk governance [91], we also suggest that international collaborations could potentially strengthen capacity building, technical assistance, and knowledge exchange on emergency response. We further suggest that future studies could prioritise data collection of BMCIs in LMICs, particularly in regions with limited emergency response infrastructure, to contribute to a more comprehensive understanding of the challenges and limitations faced in BMCI response in resource-limited settings.

### Communication

Communication was frequently identified and categorized into three types of factors: *on-scene communication*, referring to exchanges among rescuers at the incident site or with commanders and dispatch centers located on- or off-scene; *inter-agency communication*, referring to interactions between different organizations, such as police, fire rescue, and medical services; and *public informing*, a one-way communication without information exchange, such as warnings on social media or emergency broadcasts.

Inter-agency communication failures [53, 58, 69, 75, 81–83] often arose from a lack of prior collaboration between agencies, leading to confusion in resource use and decision making. For example, the 2001 WTC attack exposed inadequate communication between fire and police departments, incident command, dispatch, and hospitals, hampering management [53]. Similarly, the poor communication among responding organizations during a factory explosion in Japan, led to a situation where firefighters knew about nearby explosives while responding physicians were unaware, thus unknowingly risking their lives [82]. These examples highlight the importance of well-functioning inter-agency communication and coordination, including a-priori knowledge and familiarity to procedures and structures of agencies involved, an area that can potentially be addressed more readily than some of the other challenges in BMCI management.

Inadequate communication on-scene [20, 47, 51, 52, 69, 75, 82, 83, 86], typically resulted from miscommunication, delays in conveying incident magnitude, poor information flow among first responders and unfamiliarity with operational procedures. During the Singapore Airlines Flight 006 accident, initial information from the crash site was reported to be confusing, misleading, and lacking essential details, leading to unsuccessful information flow [47]. Similarly, in the West Pharmaceutical Services explosion, Cairn et al. noted false on-scene messages compounding communication failures [20]. Other issues included poor coordination between incident commander and MCI coordinator [69], delays in relaying the scale of the BMCI [75], and unfamiliarity with contact methods on-scene [82]. Some of these problems, such as weak command structures, can be addressed through training and clear protocols. However, initial false or delayed information remains difficult to eliminate. To improve early communication, we suggest first responders use the METHANE mnemonic (Major incident declared, Exact location, Type of incident, Hazards, Access routes, Number of casualties, and Emergency services present or needed) to structure and convey critical information swiftly and accurately [92, 93]. Its effectiveness was illustrated in 2023 when the first responders METHANE report enabled a timely and efficient escalation of response during a hospital fire in Italy [86].

### Chaos at scene

Chaos can be assumed to be an implicit part of almost any MCI, and possibly due to this, explicitly mentioned in only seven of the included articles [5, 43, 47, 48, 61, 70, 71]. Where chaos was noted, responders faced extraordinary challenges; threats and physical violence [5, 43, 61, 68], large numbers of survivors dispersing into nearby buildings in seek of shelter [48], or a BMCI amidst a typhoon rolling in [47]. But several of the identified factors can be interpreted to be at least partially a consequence of chaos on-scene, such as inadequate information provided to receiving hospitals [20, 43, 47, 54, 56–58, 62, 83], poor or absent command structures [47, 54, 57, 70, 71, 82, 83], unsatisfactory coordination or resource allocation [48, 52, 55, 58, 75, 79, 83, 86], and deviations from established protocols [44, 49, 53, 65, 69–71, 74, 78, 83]. The factor “challenging accident site” can likewise imply chaotic circumstances: people jumping from the WTC towers onto rescuers and vehicles, significant traffic congestions, ongoing fires, explosions, panic among victims, and casualty numbers exceeding 200 were identified as specially demanding [8, 43, 46, 48, 51, 53, 54, 58, 61, 65, 68, 70].

### Self-evacuation and varied transport methods

Evacuation is also an implicit part of any MCI. *Self or bystander evacuation* consistently emerged as an identified factor from the documents [5, 8, 17, 45, 47, 51, 57, 59, 61, 62, 64, 70, 71, 73], frequently overlapping with the factor “*varied non-medical transport methods to hospital*”. In many accounts, evacuation with non-medical transports was performed spontaneously by victims and bystanders in private vehicles [8, 17, 45, 47, 48, 52, 57, 59, 61, 62, 64, 70, 71], but professionals also deployed it as a response tactic [5, 42–44, 47]. Ambulance overload was used as an ad-hoc solution in three incidents [5, 70, 71].

Self or bystander evacuation appears to be a common factor in BMCIs, as people naturally tend to flee danger and bystanders assist. This is a well-known phenomenon in all types of MCIs [94–98]. For example Reilly et al. found that only 36% of disaster victims arrive to the hospital by ambulance, while 63% use other means [99].

Several articles in our study emphasized the critical importance of accounting for self-evacuation in future planning [49, 52, 59, 61]. For instance, Welling et al. noted in the Volendam café fire, that severely burned patients often remain alert and mobile long enough to evacuate themselves [49]. Waage et al., discussing the World Trade Center attack [52], Richardson and Kumar, analysing the Canberra Bushfires [59], and von Schreeb reporting on the Beslan terrorist attack [61], all emphasized the need for emergency plans to facilitate for self-evacuation and advised to use all available transport methods in the evacuation efforts.

Our findings align with these calls to facilitate for both self-evacuation and private transportation, suggesting that greater focus might be placed on sufficient and well-prepared resources at the nearby receiving hospitals. A clear example of this comes from Beslan, where authorities had days to prepare for field hospitals, command structures, and personnel, before violence erupted and ended in flames and despite preparations, patients were still mostly evacuated by family and community, uncoordinated and untriaged, causing chaos and traffic congestion on-scene.

### Traffic congestion

Traffic congestion per se, whether contextual or self-induced by emergency services, was identified as a recurring challenge [48, 53, 62, 67, 75]. In our study, traffic congestion was twice caused by uncoordinated flow of emergency responders who arrived at the site of their own volition and not as part of a coordinated effort. The most notorious example occurred during the 2001 WTC attack [52, 53], when emergency responders arrived unauthorized and unbidden, blocking all roads in a 2 km radius, causing a gridlock that lasted for two hours and prevented any ambulances from accessing or leaving the site.

To reduce the risk of self-induced gridlocks in future, it might be beneficial to raise awareness and provide suitable training for all first responders, not only incident commanders, ensuring all responders ideally understand the potential threat of traffic congestion. These congestions in MCIs are well-documented, with also technical solutions proposed to address the issue [100–102].

### Triage and response strategies

A “load and go” as response strategy was reported in five cases, often implemented ad-hoc by the responders, despite disaster plans advocating for a “stay and play” with field hospitals and on-site treatment [43, 47, 67, 79, 84]. The only reported “stay and play” scenario was the Volendam café fire, where responders consciously chose to establish on-site treatment due to geographical constraints [48].

Several articles discussed triage as part of their response [8, 22, 44, 49, 56–58, 65, 67, 70, 71, 73–75, 78, 82–84, 86], highlighting issues such as incorrect triage [58], overtriage [83], and simultaneous use of different triage systems [74]. Two articles identified Total Body Surface Area (TBSA) estimation as problematic, advocating against its use in future BMCI triage protocols [49, 78]. Specific triage protocols also demonstrated limitations, such as The Simple Triage and Rapid Treatment (START) protocol, that failed to categorize patients correctly due to underlying neurological disorders [86], or inhalation injuries being under-triaged, leading to unexpected patient deterioration and requiring ad-hoc protocol deviations from the START protocol [78].

In-hospital BMCIs posed additional challenges for the triage. Three of five in-hospital fires in this study reported triage errors related to underlying comorbidities, which not only complicated medical categorization but also delayed evacuations and required extensive rescue resources [74, 75, 83]. This placed a significant physical and psychological burden on emergency responders encountering these situations [86].

These findings underscore the difficulty of triaging patients with burns, especially with underlying medical conditions, and support our earlier conclusion that capacity for sudden patient surges in the receiving hospitals is critical, as responders may deviate from disaster plans and default to rapid evacuations.

### In-hospital fires, industrial environments and CBRNE threats

Another identified factor with in-hospital fires was the inaccessibility of electronic records, prompting calls for physical charts or tags at bedsides [69, 74, 83]. Besides this, in-hospital fires pose potential chemical, biological, radiological, nuclear, and explosive (CBRNE) threats, with the possible presence of infectious materials, radiation material, and pressurized gases at the accident sites [75].

Similar risks extend to industrial environments, where hazardous materials are often present. Five of the included BMCIs were not only BMCIs but also CBRNE accidents; four involved a chemical dimension, while one had chemical, biological and radiological dimensions [22, 63, 72, 75, 82]. All of these accidents failed to handle the CBRNE dimension, including insufficient training and inadequate patient decontamination. Both responders and victims were compromised by poor education and situational awareness. As four of these incidents occurred in factories, we suggest that any industrial setting as an accident scene could warrant CBRNE considerations, and that it may be beneficial for all first responders to have a basic understanding of CBRNE hazards.

### Concluding remarks

Lastly, for the improvement of future management of BMCIs, we suggest that all 71 identified factors, presented in Table 5, be inspected as a whole and individually. We wish to emphasize that a reported factor itself may carry greater intrinsic value regardless of whether it was reported as positive or negative. The positive or negative nature of an observation is subjective and can vary, and the positivity or negativity has thus not been discussed.

## Limitations

The first limitation we wish to highlight is qualitative research per se and interpretation bias. How something is understood, often depends on the perspective of the person observing it. For example, Lee et al. described the management of the Singapore Airlines Flight 006 accident mostly as a failure, while Pesola et al. described the exact same actions in the same accident, as positive and helpful [103]. This difference in viewpoints highlights also the subjective nature of qualitative studies and points to the possibility of interpretation bias also in our review. Secondly, using a single analyst can provide consistency and deeper immersion in the thematic analysis, supporting intra-rater reliability. However, a single analyst can simultaneously be a methodological limitation by potentially introducing interpretative bias or by limiting inter-rater reliability.

The developed search strategy may also in hindsight have been too narrow, as we identified no documents describing the pre-hospital management of the 2015 Romania nightclub fire or the 2017 Grenfell Tower fire, that were both widely reported in the news internationally. Such omissions highlight potential gaps either in the academic literature or restrictions in our search parameters. A geographical and publication bias exists, as many large-scale MCIs, including BMCIs, occur in low- and middle-income countries. Since LMICs typically produce fewer scientific publications than high-income countries, their experiences are likely underrepresented in this review. As a result, our findings primarily reflect high-income settings, limiting their applicability.

As for the planned inclusion criteria, we diverted from the initial protocol, when after consultation of experts included all KAMEDO reports published after 2001 for screening. These reports are special publications produced by the Swedish Civil Contingencies Agency that analyse global disaster responses, highlighting lessons learned from these incidents. A second diversion happened when one article was identified for inclusion after classifying it as a BMCI in full text review. Moreover, a quality assessment using a predefined checklist based on the Oxford Centre of Evidence-based Medicine (CEBM) Levels of Evidence [104], could have helped evaluate the robustness of each included document and classify the reliability of findings. Finally, while excluding war focused documents may reduce confounding factors (e.g., massive resources unique to military settings), it also limits civilian preparedness strategies that might otherwise benefit from military innovations.

## Conclusion

A rapid emergency response was a commonly identified factor in high-income countries, while significant disparities were noted in low-income countries. A crucial finding was the need to include self-evacuation in disaster plans and facilitate patient evacuation with various non-medical transport methods. Potentially preventable issues included traffic congestion, poor inter-agency communication, and a lack of CBRNE knowledge among first responders. In-hospital BMCI and BMCIs with over 200 casualties were identified as specially demanding.

Each BMCI is a unique event, and thus each factor reported in a previous BMCI may hold significance and is potentially worthy of consideration in future preparedness and response efforts. We therefore suggest reviewing all identified factors carefully, regardless of their frequency or type, to determine their potential relevance.

## Supplementary Information

Supplementary file 1

Supplementary file 2

Supplementary file 3

## Data Availability

The data extracted, used and analysed during the current study are available from the corresponding author on reasonable request.

## Abbreviations

BLEVE: Boiling liquid expanding vapor explosion
BMCI: Burn mass casualty incident
CBRNE: Chemical, biological, radiological, nuclear, and explosive
CINAHL: Cumulative index to nursing and allied health literature
Embase: Excerpta medica database
EMT: Emergency medical technician
HIC: High-income country
HEMS: Helicopter emergency medical service
LMCI: Low- and middle-income countries
MCI: Mass casualty incident
METHANE: Major incident declared, exact location, type of incident, hazards, access routes, number of casualties, and emergency services present or needed
PCC: Population, concept, context
PRISMA-ScR: Preferred reporting items for systematic reviews and meta-analyses extension for scoping reviews
START: Simple triage and rapid treatment
TBSA: Total body surface area
WTC: World trade center
