# Supplementary material for: Factors influencing the pre-hospital management of civilian burn mass casualty incidents in the 21st century: a scoping review
Source: Scand J Trauma Resusc Emerg Med. 2025 May 1;33:74. doi: 10.1186/s13049-025-01380-9 (PMC12044938; doi:10.1186/s13049-025-01380-9)
Supplement: Supplementary file 1 [file 13049_2025_1380_MOESM1_ESM.docx]

**Supplementary file 1. Search strategies**

**Table 7.** The search strategy used for CINAHL.

| **CINAHL** |
| --- |
| (MH "Burns") OR (TI burns OR AB burns) **AND** (MH "Disasters" OR MH "Mass Casualty Incidents") OR (TI "mass casualty incidents" OR AB "mass casualty incidents" OR TI disasters OR AB disasters) **AND** (MH "Emergency Medical Services" OR MH "First Aid" OR MH "Triage" OR MH "Prehospital Care") OR (TI "prehospital care" OR AB "prehospital care" OR TI "emergency medical services" OR AB "emergency medical services" OR TI triage OR AB triage OR TI "first aid" OR AB "first aid") |

**Table 8.** The search strategy used for Cochrane Library.

| **Cochrane Library** |
| --- |
| ("burns") **AND** ("mass casualty incidents" OR "disasters" OR "disaster medicine") **AND** ("prehospital care" OR "emergency medical services" OR "first aid" OR "triage" OR "emergency treatment") |

**Table 9.** The search strategy used for Google Scholar.

| **Google Scholar** |
| --- |
| (burns) **AND** ("mass casualty incidents" OR disasters OR "disaster medicine") **AND** ("prehospital care" OR "emergency medical services" OR "first aid" OR triage OR "emergency treatment") -guidelines -simulation -training |

**Table 10.** The search strategy used for Embase.

| **Embase** |
| --- |
| (MH "Burns") OR (TI burns OR AB burns) **AND** (MH "Disasters" OR MH "Mass Casualty Incidents") OR (TI "mass casualty incidents" OR AB "mass casualty incidents" OR TI disasters OR AB disasters) **AND** (MH "Emergency Medical Services" OR MH "First Aid" OR MH "Triage" OR MH "Prehospital Care") OR (TI "prehospital care" OR AB "prehospital care" OR TI "emergency medical services" OR AB "emergency medical services" OR TI triage OR AB triage OR TI "first aid" OR AB "first aid") |

**Table 11.** The search strategy used for PsycINFO.

| **PsycINFO** |
| --- |
| Burns" OR "Thermal Injuries") **AND** ("Mass Casualties" OR "Disasters" OR "Disaster Psychology" OR "Mass Casualty Incidents" OR "Disaster Medicine") **AND** ("Emergency Medical Services" OR "Prehospital Care" OR "First Aid" OR "Triage" OR "Emergency Treatment" OR "Psychological First Aid") |

**Table 12.** The search strategy used for PubMed.

| **PubMed** |
| --- |
| (Burns[MeSH Terms] OR Burns[Text Word]) **AND** ("Mass Casualty Incidents"[MeSH Terms] OR Disasters[MeSH Terms] OR "Mass Casualty Incidents"[Text Word] OR "Disaster Medicine"[MeSH Terms]) **AND** ("Prehospital Emergency Care"[MeSH Terms] OR "Emergency Medical Services"[MeSH Terms] OR "First Aid"[MeSH Terms] OR Triage[MeSH Terms] OR "Emergency Treatment"[Text Word]) |

**Table 13.** The search strategy used for Web of Science.

| **Web of Science** |
| --- |
| TS=(Burns) **AND** TS=(("Mass Casualty Incidents" OR Disasters OR "Mass Casualty" OR "Disaster Medicine")) **AND** TS=(("Pre-hospital Management" OR "Emergency Medical Services" OR "First Aid" OR Triage OR "Emergency Treatment")) |

*Tables 6-12 show the used search strategies for the databases in question. All search strategies used the limitation of January 1^st^ 2001, to April 1^st^ 2024.*
