# Supplementary material for: Factors influencing the pre-hospital management of civilian burn mass casualty incidents in the 21st century: a scoping review
Source: Scand J Trauma Resusc Emerg Med. 2025 May 1;33:74. doi: 10.1186/s13049-025-01380-9 (PMC12044938; doi:10.1186/s13049-025-01380-9)
Supplement: Supplementary file 2 [file 13049_2025_1380_MOESM2_ESM.docx]

**Supplementary file 2. Template used for data extraction**

**Table 14.** The template used for data extraction in Covidence.

| **Template for data extraction** | |
| --- | --- |
| **General information** | |
| Title of publication  Journal / Where published  Author(s)  The authors' affiliation to the incident | Year of publication  Type of publication  Origin/country of origin where the study was published or conducted |
| **Aim, methods and key findings** | |
| Aim/purpose of the publication  Study design/methodology | Key findings |
| **Incident information** | |
| Number of incidents reported in the publication  Year of incident(s)  Type of incident(s)  Short description of incident(s)  Name of incident(s)  Country of incident(s) | Number of cases or casualties  On scene deaths / mortality rate  Type and severity of burns in patients evacuated  Type of triage used  Responding emergency services |
| **Research questions** | |
| - Factors reported as having a positive effect, including reported achievements and successes, that influenced the pre-hospital management.  - Factors reported as having a negative effect, as well as reported failures and obstacles, that impacted the pre-hospital management. | - Recommendations made for future incidents regarding subjects identified in point 20.-21.  - Other factors reported or stated to influence the situation at hand. |
| **Additional notes** | |
| Other remarks or additional comments |  |

*Table* *14* *presents all data that was extracted from the included documents. Not all extracted data was used in this review.*
