# Supplementary material for: Factors influencing the pre-hospital management of civilian burn mass casualty incidents in the 21st century: a scoping review
Source: Scand J Trauma Resusc Emerg Med. 2025 May 1;33:74. doi: 10.1186/s13049-025-01380-9 (PMC12044938; doi:10.1186/s13049-025-01380-9)
Supplement: Supplementary file 3 [file 13049_2025_1380_MOESM3_ESM.docx]

**Supplementary file 3. Included articles**

**Table 15.** All the included studies in the review.

| Author(s) | Year | Study location | Study type | Title | Journal / Where published |
| --- | --- | --- | --- | --- | --- |
| Hadac (42) | 2002 | Czech Republic | Case report | Ensuring prehospital care in thermal injuries: experience from an explosion in a textile factory | Acta Chirurgiae Plasticae |
| Gewalli and  Fogdestam (43) | 2003 | Sweden | Case report and retrospective analysis | Triage and Initial Treatment of Burns in the Gothenburg Fire Disaster 1998: On-Call Plastic Surgeons' Experiences and Lessons Learned | Scandinavian Journal of Plastic and Reconstructive Surgery and Hand Surgery |
| Cassuto and  Tarnow (44) | 2003 | Sweden | Case study and retrospective analyse | The discotheque fire in Gothenburg 1998  A tragedy among teenagers | Burns |
| Riddez and Dellgar (45) | 2005 | Sweden | Special report | KAMEDO Report No. 75 Fire Catastrophe in Gothenburg 29-30 October 1998 | Prehospital and Disaster Medicine |
| Al-Hoqail (46) | 2004 | Saudi Arabia | Cross-sectional study | A fire disaster at a wedding in a village in the Eastern province, Saudi Arabia | Annals of Burns and Fire Disasters |
| Björnhagen et al. (47) | 2006 | Sweden | Special report | KAMEDO Report NO. 82 Explosion at the Fireworks Warehouse in the Netherlands in 2000 | Prehospital and Disaster Medicine |
| Lee et al. (48) | 2001 | Taiwan | Incident report | Emergency Medical Preparedness and Response to a Singapore Airliner Crash | Academic Emergency Medicine |
| Welling et al. (49) | 2005 | The Netherlands | Case report | The café fire on New Year’s Eve in Volendam, the Netherlands: description of events | Burns |
| Welling et al. (50) | 2007 | The Netherlands | Observational study | Reliability of the Primary Triage Process After the Volendam Fire Disaster | The Journal of Emergency Medicine |
| Roche (51) | 2001 | USA | Personal commentary | Eyewitness to Terror: EMS Responds to the Attack on the Pentagon | Air Medical Journal |
| Wang et al. (52) | 2005 | USA | Case report and retrospective review | The Pentagon and 9/11 | Critical Care Medicine |
| Waage et al. (53) | 2006 | Sweden | Special report | KAMEDO Report No. 84 Terrorist Attacks Against the World Trade Center, 11 September 2001 | Prehospital and Disaster Medicine |
| Pryor et al. (54) | 2009 | USA | Retrospective observational study with elements of a case report. | The 2001 World Trade Center Disaster: Summary and Evaluation of Experiences | European Journal of Trauma and Emergency Surgery |
| Brolen et al. (55) | 2007 | Sweden | Special report | KAMEDO Report 89: Terrorist Attack in Bali, 2002 | Prehospital and Disaster Medicine |
| Tran et al. (56) | 2003 | Australia | Case report and retrospective analyses | The Bali bombing: civilian aeromedical evacuation | The Medical Journal of Australia |
| Dacey (57) | 2003 | USA | Perspective | Tragedy and Response — The Rhode Island Nightclub Fire | The New England Journal of Medicine |
| Gutman et al. (58) | 2003 | USA | Case report and retrospective analyses | The Station Nightclub Fire and Disaster Preparedness in Rhode Island | Medicine and Health Rhode Island |
| Harrington et al. (59) | 2005 | USA | Case report and retrospective analyses | The Station Nightclub Fire | Journal of Burn Care & Rehabilitation |
| Richardson and Kumar (60) | 2004 | (60) | Case report | Emergency Response to the Canberra Bushfires | Medical Journal of Australia |
| Cairns et al. (61) | 2005 | USA | Case report and retrospective analyses | Managing a Combined Burn Trauma Disaster in the Post-9/11 World: Lessons Learned from the 2003 West Pharmaceutical Plant Explosion | Journal of Burn Care & Rehabilitation |
| Vilke et al. (62) | 2006 | USA | Case report and retrospective cohort study | Impact of the San Diego County Firestorm on Emergency Medical Services | Prehospital Disaster Medicine |
| Von Schreeb et al. (63) | 2004 | Sweden | Case report and retrospective analyses | Emergency Care following the Terrorist Attack in Beslan, North Ossetia, Russian Federation, 2004 | International Journal of Disaster Medicine |
| Umer et al. (64) | 2009 | Pakistan | Case report and analyses of outcome of injuries | Suicide bombings: process of care of mass casualties in the developing world | Disasters |
| Ma et al. (65) | 2007 | China | Retrospective cohort study | Mass chemical burn casualty: Emergency management of 118 patients with alkali burn during a Matsa typhoon attack | Burns |
| Young et al (66) | 2009 | USA | Analytical report | Double Fire Tragedy of Kenya | Eplasty - Open access Journal of Plastic Surgery |
| Cameron et al. (67) | 2009 | Australia | Case study and retrospective analysis | Black Saturday: the immediate impact of the February 2009 bushfires in Victoria, Australia | Medical Journal of Australia |
| Seifman et al. (29) | 2011 | Australia | Case report and retrospective analyses | Bushfire Disaster Burn Casualty Management: The Australian “Black Saturday” Bushfire Experience | Annals of Plastic Surgery |
| Greenhalgh et al. (68) | 2012 | USA | Case report | The ABC Daycare Disaster of Hermosillo, Mexico | Journal of Burn Care & Research |
| Kearns et al. (69) | 2014 | USA | Case report and retrospective analyses | Hospital Bioterrorism Planning and Burn Surge | Biosecurity and Bioterrorism: Biodefense Strategy, Practice, and Science |
| Picazo et al. (70) | 2015 | Chile | Incident report | Prison Fire in Santiago de Chile | www.majorincident-reporting.net |
| Koning et al. (71) | 2014 | The Netherlands | Case report and retrospective analyses | Indoor fire in a nursing home: evaluation of the medical response to a mass casualty incident based on a standardized protocol | European Journal of Trauma and Emergency Surgery |
| Kumar et al. (17) | 2013 | India | Case report | Fire disaster following LPG tanker explosion at Chala in Kannur (Kerala, India): August 27, 2012 | Burns |
| Dal Ponte et al. (73) | 2015 | Brazil and USA | Case report and retrospective analyses | Mass-casualty Response to the Kiss Nightclub in Santa Maria, Brazil | Prehospital and Disaster Medicine |
| Heltne (74) | 2015 | Norway | Incident report | Truck and tunnel fire | www.majorincident-reporting.net |
| Zhang et al. (75) | 2015 | China | Case report | Mass chemical casualties: Treatment of 41 patients with burns by anhydrous ammonia | Burns |
| Zhang et al. (76) | 2015 | China | Case report and retrospective analyses | Injuries following a serious hydrofluoric acid leak: First aid and lessons | Burns |
| Hang, Jianan and Chunmao (77) | 2016 | China | Case report and descriptive study | Experience in managing an urban massive burn incident: The Hangzhou bus attack on 5 July 2014 | Burns |
| Jang et al. (78) | 2020 | South Korea | Case report | Disaster Response to a Mass Casualty Incident in a Hospital Fire by Regional Disaster Medical Assistance Team: Characteristics of Hospital Fire | Disaster Medicine and Public Health Preparedness |
| Roman-Morales et al. (79) | 2016 | Mexico | Incident report | Gas explosion | Majorincidentreporting.net |
| Wang and Lin (80) | 2015 | Taiwan | Perspective | Responding to mass burn casualties caused by corn powder at the Formosa Water Park in 2015 | Journal of the Formosan Medical Association |
| Yang and Shih (81) | 2016 | Taiwan | Empirical research article | A Coordinated Emergency Response: A Color Dust Explosion at a 2015 Concert in Taiwan | Public Health Practice |
| Wang et al. (8) | 2016 | Taiwan | Retrospective cohort study | Experience of distributing 499 burn casualties of the June 28, 2015 Formosa Color Dust Explosion in Taiwan | Burns |
| Lin et al. (83) | 2019 | Taiwan | Case report and retrospective analyses | Challenges of Burn Mass Casualty Incidents in the Prehospital Setting: Lessons From the Formosa Fun Coast Park Color Party | Prehospital Emergency Care |
| Lin et al. (84) | 2021 | Taiwan | Quality Improvement Study | An innovative emergency transportation scenario for mass casualty incident management Lessons learnt from the Formosa Fun Color Dust explosion | Medicine |
| Lin et al. (85) | 2022 | Taiwan | Original article / Retrospective cohort study | Patients’ survival rates and their correlated factors in the prehospital setting of a dust explosion incident | Hong Kong Journal of Emergency Medicine |
| Wang et al. (86) | Original article | Taiwan | Original article / Case report and retrospective analyses | Prehospital Scheduling Management by the Critical Path Method for Burn Mass Casualty Incidents | The Journal of Health Care Organization, and Financing |
| Kondo et al. (87) | 2019 | Japan | Case report and retrospective analyses | Multiple Patients With Burn Injury Induced by a Chemical Explosion Managed by Physician-Staffed Helicopters | Disaster Medicine and Public Health Preparedness |
| Choi et al. (88) | 2022 | South Korea | Field report / Case report | Analysis of Disaster Medical Response: The Sejong Hospital Fire | Prehospital and Disaster Medicine |
| Gamberini et al. (89) | 2021 | Italy | Retrospective analyses | Mass Casualty Management After a Boiling Liquid Vapor Explosion in an Urban area | The Journal of Emergency Medicine |
| Shao et al. (90) | 2023 | China | Original article / Case report | Triage and Evaluation of Blast-Injured Patients in Wenling Liquefied Petroleum Gas Tanker Explosion | Journal of Burn Case & Research |
| Ripoll-Gallardo et al. (91) | 2023 | Italy | Report from the Field / Case report | Prehospital Mass Casualty Incident Response to a Fire in a Nursing Home in Milan, Italy: Actions Taken and Shortcomings | Disaster Medicine and Public Health Preparedness |

*Table 15 presents all the included documents in this review, where they were published, what type of documents they were and what they reported on.*
